# Prof. of Physiology in Oglethorpe Medical College

**Published:** 1859-01

**Authors:** 


### PROF. OF PHYSIOLOGY IN OGLETHORPE MEDICAL COLLEGE.

A. G. Thomas, A. M., M. D., of this city, a gentleman of decided talents and fine scientific and Literary attainments has been appointed Professor of Physiology, &c., in the Oglethorpe Medical College, of Savannah, Georgia.

We regard the Institution as fortunate in securing his services.

